# Exhaled volatile organic compounds in the detection of colorectal cancer: a systematic review and meta-analysis

**DOI:** 10.17179/excli2024-7042

**Published:** 2024-05-17

**Authors:** Daniah Alsaadi, Nicolle Clements, Natiya Gabuniya, Nader Francis, Manish Chand

**Affiliations:** 1 The Division of Surgery and Interventional Science, University College London, London, United Kingdom; 2 Clinical Research Facility Galway, Galway University Hospital, National University of Ireland, Galway, Republic of Ireland; 3 Plastic and Reconstructive Surgery Department, Guy's and St Thomas' Hospital, London, United Kingdom; 4 Department of General Surgery, Yeovil District Hospital NHS Foundation Trust, Yeovil, United Kingdom

**Keywords:** colorectal cancer, volatile organic compounds, exhaled, breath analysis

## Abstract

There is an apparent need for novel non-invasive colorectal cancer (CRC) screening tests that are more acceptable to patients and can reliably detect CRC or reduce the number of unnecessary colonoscopies performed in cancer-free patients. An emerging number of studies demonstrate the potential value of exhaled volatile organic compounds (VOCs) as a diagnostic and triaging test for CRC. A systematic appraisal and meta-analysis of the published evidence was done to determine whether exhaled VOCs can be used in the detection and screening of CRC. Nine electronic databases were searched from inception of the databases until August 2020. Quantitative and descriptive data of CRC patients and healthy control (HC) participants who underwent VOCs breath analysis was extracted. In addition, where possible, sampling methods, analytical platforms, processors, and specific breath biomarkers found in each study were recorded. Fourteen articles were included in the systematic review with 491 colorectal patients and 754 HC participants (n=1245). Sub-group meta-analysis was conducted on nine of those articles and the pooled sensitivity was estimated to be 0.89 (95 % CI = 0.80-0.99) whereas specificity was 0.83 (95 % CI = 0.74-0.92). Heterogeneity of pooled sensitivity and specificity was estimated as I^2^=11.11 %. Although this study was limited by small sample size and different analytical platforms, the proposed future framework resolves such limitations and standardizes future research. It is reasonable to deduce that VOCs breath analysis is certainly a field of research that can progress to replace traditional methods within the framework of CRC screening and diagnosis.

## Introduction

Colorectal cancer (CRC) is reported as the third most commonly diagnosed cancer and the second leading cause of cancer-related deaths worldwide (Rawla et al., 2019[16]). Despite the introduction of colorectal screening programs, patient compliance has remained low worldwide (Wools et al., 2016[23]). Colonoscopy remains the gold-standard investigative approach for the screening and diagnosing of CRC (CDC, 2020[4]). Complications, such as bleeding and perforation, as well as the necessity for bowel preparation are commonly weighed against the result, i.e. primarily reassurance (Rees et al., 2016[18]). In 2018, The United States (US) Behavioural Risk Factor Surveillance System stated that approximately 21.7 million adults aged 50-75 years have never been screened for CRC (CDC, 2020[5]). Some screening programs employ the use of faeces-based non-invasive tests prior to conducting a colonoscopy. The current fast-track colonoscopy referral pathway in the UK puts forward approximately 300,000 suspected patients for screening annually (Thornton et al., 2016[20]). Due to the non-specific nature of CRC symptoms, the majority of these patients are screened via colonoscopy within two weeks and only 3 %-10 % are diagnosed with CRC. Consequently, there is a need for novel non-invasive CRC screening tests that are more acceptable to patients and that can reliably detect CRC or reduce the number of unnecessary colonoscopies performed in cancer-free patients. 

Theoretically, different diseases can be categorized by unique metabolomic profiles (Hanna et al., 2019[10]). Volatile organic compounds (VOCs; carbon-based compounds that mainly comprise alkanes, benzenes and aldehydes) are a subtype of metabolites that are linked to oxidative stress and cell-membrane peroxidation. Reference to VOCs in exhaled breath was first made by Pauling and his colleagues in 1971 (Pauling et al., 1971[14]). They can be detected in several types of biological samples such as faeces, urine, serum, sputum and in the breath. These carbon compounds can be affected by both internal and external factors such as smoking (Reade, 2016[17]). Cancer is a metabolic disease that uses cellular metabolic changes to maintain a high rate of proliferation. Gene mutations and protein changes in the tumor cells lead to an oxygenation of the polyunsaturated fatty acids in the cell membranes and thus to a change in the VOCs in cancer patients (Altomare et al., 2016[2]). Although the detection of disease through breath analysis is an emergent field of research, it can be traced back to approximately 44 BC. Hippocrates taught his students to smell the breath of their patients and to pour human saliva on hot coals to identify their ailments (Reade, 2016[17]). In the 1980s, urea breath tests began to be used clinically in the diagnosis of *Helicobacter pylori*-related gastritis (Graham and Miftahussurur, 2018[9]). 

The current paper considers breath analysis for the detection of CRC, as its painless and non-invasive nature makes it a favorable method that will increase patient compliance in the future. A review of the current literature indicated that the bulk of the available literature instead considered VOCs without a focus on a specific cancer subtype. Studies that did report specific data for CRC and exhaled VOCs were small in size. Recognizing that these studies may individually represent limited data for impacting clinical practice on their own and considering that no diagnostic meta-analysis or systematic review had previously been conducted to integrate these data and derive relevant conclusions, a systematic appraisal and meta-analysis of the published evidence was conducted in the current paper to determine whether the analysis of VOCs in the breath could enable the detection of CRC and whether it held value as a non-invasive screening method. 

## Materials and Methods

### Guidelines 

The Preferred Reporting Items for Systematic Review and Meta-Analyses (PRISMA) guidelines were followed while conducting this study and while reporting this systematic review. Concurrently, the Meta-analysis of Observational Studies in Epidemiology (MOOSE) guidelines were followed to ensure the highest possible meta-analysis quality. The PRISMA and MOOSE checklists are included in the supplementary information, Appendix A. Obtaining patient consent and approval from a research ethics committee was not required to conduct this study because this systematic review and meta-analysis were based on previously published studies. Nonetheless, established ethical standards were followed. This manuscript received no funding, has no conflict of interests to disclose and was not registered. 

### Search strategy 

The search strategy was validated by a librarian (JS) at University College London (UCL). The search was performed independently by two investigators (DA and NC). Nine electronic databases were searched starting at their inception date, i.e. Proquest, Pubplus, Cochrane, Google Scholar, Ovid Embase Classic + Embase, Ovid MEDLINE and In-Process and Other Non-Indexed Citations, Ovid MEDLINE, the US National Library of Medicine database (Pubmed) and Scirus. Furthermore, the search was supplemented using the UCL explore library (which included 719 databases). The initial search was conducted on 15 July 2020 and repeated on 15 August 2020 to identify additional literature published since the first search. The full list of search strings utilized is reported in the supplementary information, Appendix B.

### Eligibility criteria 

All relevant articles were retrieved without geographic, size, date, or outcome limitations. Eligibility criteria included: (1) articles that included segregated quantitative data on patients with CRC; (2) studies that included analyzed VOCs, particularly within the exhaled breath of participants for diagnosing CRC; (3) studies were written in the English language; (4) peer-reviewed journal articles, conference abstracts and posters with sufficient segregated data; (5) studies must have reported at least one or more of the following types of quantitative data: sensitivity, specificity, accuracy, recognition, true positive (TP), false negative (FN), false positive (FP), true negative (TN), number of CRC and healthy control (HC) participants and characteristic and demographic factors including age, smoking and gender. Papers were excluded if: (1) studies were performed on non-human subjects; (2) the patients studied had secondary CRC or surveillance; (3) studies that failed to provide any quantitative data. Meta-analysis criteria differed slightly in that both sensitivity and specificity had to have been reported in addition to the number of CRC patients and those in the HC group.

### Study screening

Mendeley was used for screening. The process of screening was conducted independently by the same investigators previously noted (DA and NC). An initial article title screen was conducted within all databases during the electronic search to eliminate obviously irrelevant articles. They were then imported into a joint Mendeley account by both assessors. Next, the two reviewers independently conducted a secondary title screen, which was followed by an abstract screen and a final full-text screen as per the previously determined eligibility criteria. Screening decisions were then cross-matched, and any discrepancies were resolved during a panel discussion with a third and fourth reviewer (SN and CP) and a consensus was achieved.

### Descriptive and methodologic analysis

The extracted data were categorized according to factors that could potentially influence exhaled VOC levels. A list of all data extracted can be found in the supplementary information, Appendix C. These aspects were classified into the following domains: (1) patient selection criteria for the CRC and HC group; (2) patient characteristics and demographic factors including gender, smoking and CRC stage; (3) the sampling and collection methods; (4) the analytical platforms and processes used; (5) other specific variability sources. 

### Quality analysis 

The Quality Assessment of Diagnostic Accuracy Studies-2 (QUADAS-2) tool was used to assess the risk of bias and applicability and quality of the articles included in this review. Additionally, the Standards for Reporting of Diagnostic Accuracy Studies (STARD) checklist was used to ensure the full transparency and completeness of the studies. 

### Statistical analysis 

The purpose of conducting a meta-analysis was to assess the strength of the evidence found in these articles; this was done by combining their results to derive a pooled weighted estimate with a higher statistical power. Articles that provided both sensitivity and specificity were analyzed using two mathematical methods. The first approach calculated the overall mean of the sensitivities and specificities stated in the respective studies. The second approach involved the retrograde mathematical calculation of the given sensitivities and specificities to obtain the TP, TN, FP and FN of each study and the resulting pooled sensitivity and specificity. The Positive Predictive Value (PPV) and Negative Predictive Value (NPV) of each study were also determined using the TP, TN, FP and FN obtained from the retrograde calculations. The confidence interval (CI) for the TP, TN, FP, FN, PPV and the NPV of each study were obtained using the simple asymptotic with continuity correction formula. In addition, the percentage of the variability that was due to heterogeneity was calculated using the quantifying inconsistency formula I^2 ^= 100 % × (Q - df)/Q, where Q and df are the Cochran chi-squared statistic and degrees of freedom, respectively. A forest plot of the sensitivity and specificity was created to illustrate the results obtained in this study.

## Results

### Literature search 

**Figure 1:**
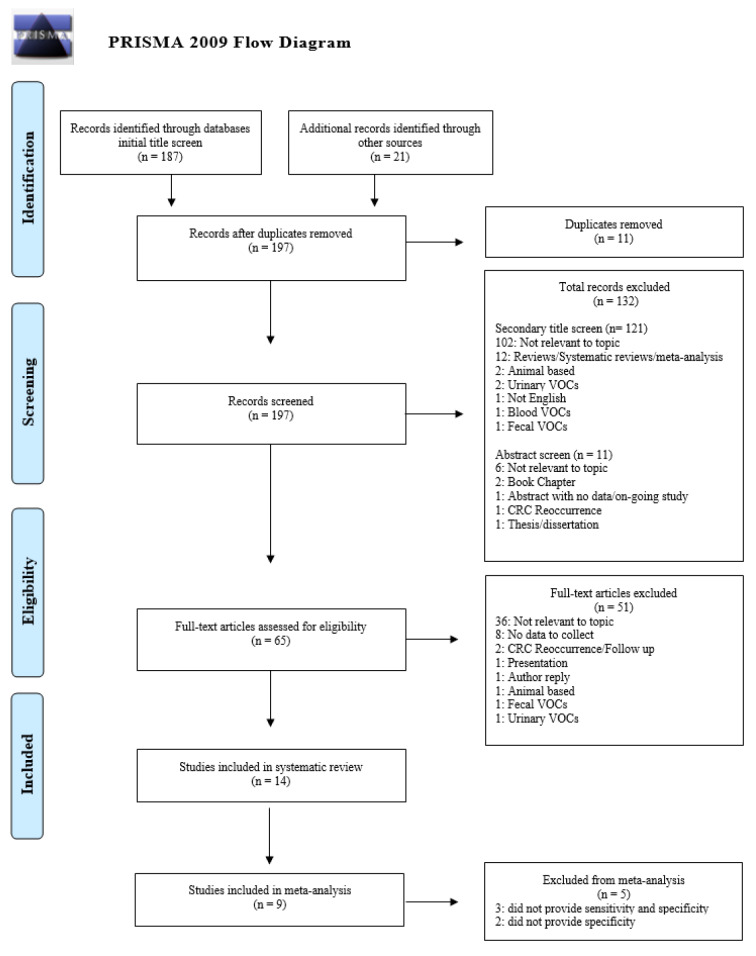
PRISMA flow diagram

Following the initial title screening of the nine databases and the UCL explore library database, 208 articles were imported into Mendeley; 197 articles were assessed in the secondary title screening after 11 duplicates were removed. A total of 121 articles were excluded after the secondary title screening for reasons including the following: irrelevance (102 articles); the type of paper (12 articles); language (one article); animal study (two articles); other types of VOCs (four articles). Subsequently, the abstract screening eliminated an additional 11 papers for reasons including the following: irrelevance (six articles); type of paper (three articles); CRC reoccurrence (one article); no quantitative data (one article). The remaining 65 articles were screened for eligibility as per the inclusion and exclusion criteria, leading to the exclusion of 51 articles for reasons including the following: irrelevance (36 articles); included no quantitative data (eight articles); other types of VOCs (two articles); type of study (two articles); animal study (one article); CRC reoccurrence and follow up (two articles). Hence, this systematic review included a total of 14 articles that passed our eligibility criteria in the final full-text screening (Altomare et al., 2013[1], 2016[2]; Amal et al., 2016[3]; Depalma et al., 2014[7]; De Vietro et al., 2020[6]; Di Lena et al., 2012[8]; Leja et al., 2015[11]: Markar et al., 2019[12]; Nakhleh et al., 2017[13]; Peng et al., 2010[15]; Sonoda et al., 2011[19]; Van Keulen et al., 2020[21]; Wang et al., 2014[22]; Zambrana et al., 2012[24]). Among the included articles, nine were included in the meta-analysis because they provided both sensitivity and specificity values (Altomare et al., 2013[1], 2016[2]; Amal et al., 2016[3]; Di Lena et al., 2012[8]; Leja et al., 2015[11]; Markar et al., 2019[12]; Sonoda et al., 2011[19]; Van Keulen et al., 2020[21]; Zambrana et al., 2012[24]). A PRISMA flow diagram summarizing the systematic search results and the screening process is illustrated in Figure 1(Fig. 1). 

### The overall characteristics of the selected studies and quality assessment 

**Figure 2:**
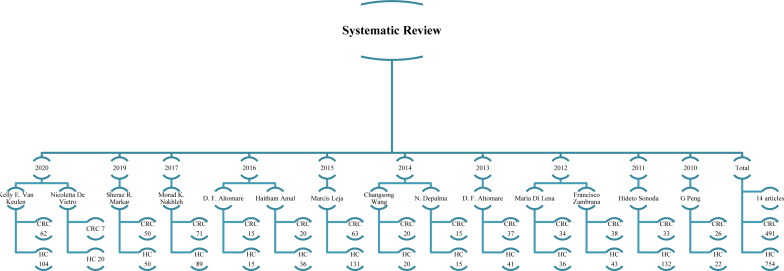
Included articles and number of CRC and HC participants CRC:Colorectal cancer; HC: Healthy Control

As shown in Table 1(Tab. 1) (References in Table 1: Altomare et al., 2013[1], 2016[2]; Amal et al., 2016[3]; De Vietro et al., 2020[6]; Depalma et al., 2014[7]; Di Lena et al., 2012[8]; Leja et al., 2015[11]; Markar et al., 2019[12]; Nakhleh et al., 2017[13]; Peng et al., 2010[15]; Sonoda et al., 2011[19]; Van Keulen et al., 2020[21]; Wang et al., 2014[22]; Zambrana et al., 2012[24]), the articles were published between the years of 2010 and 2020, 9 were based in Europe, while 5 were carried out in West and East Asia. In total, 1,416 participants were assessed, 552 of whom were CRC patients and 864 represented the HC group. However, further inspection of the studies revealed that only 491 CRC patients and 754 HC participants had been sampled for VOC testing. A diagram of the included articles and the number of CRC and HC participants is shown in Figure 2(Fig. 2) (References in Figure 2: Altomare et al., 2013[1], 2016[2]; Amal et al., 2016[3]; De Vietro et al., 2020[6]; Depalma et al., 2014[7]; Di Lena et al., 2012[8]; Leja et al., 2015[11]; Markar et al., 2019[12]; Nakhleh et al., 2017[13]; Peng et al., 2010[15]; Sonoda et al., 2011[19]; Van Keulen et al., 2020[21]; Wang et al., 2014[22]; Zambrana et al., 2012[24]). The mean age of the CRC subjects was 65.62 (58-71) years, although six of the included articles failed to provide participant ages (Depalma et al., 2014[7]; Di Lena et al., 2012[8]; Leja et al., 2015[11]; Markar et al., 2019[12]: Peng et al., 2010[15]; Zambrana et al., 2012[24]). Among the CRC cohort, the number of male patients was higher (n = 216) compared with the female group (n = 152); however, the gender of 184 CRC patients was unreported. Four studies used the American Joint Committee on Cancer's 0-IV full staging system (Amal et al., 2016[3]; Markar et al., 2019[12]; Sonoda et al., 2011[19]; Zambrana et al., 2012[24]) whereas stage 0 (carcinoma in situ) was not included in five of the other studies (Altomare et al., 2013[1], 2016[2]; Peng et al., 2010[15]; Van Keulen et al., 2020[21]; Wang et al., 2014[22]). The remaining five studies did not provide staging details but did confirm that the patients had CRC, either by colonoscopy or histology (Depalma et al., 2014[7]; Di Lena et al., 2012[8]; Leja et al., 2015[11]; Nakhleh et al., 2017[13]). Only 93 CRC patients were declared as current or ex-smokers and 276 participants had never smoked; six articles did not disclose smoking status (n = 183) (Altomare et al., 2013[1]; Depalma et al., 2014[7]; De Vietro et al., 2020[6]; Leja et al., 2015[11]; Van Keulen et al., 2020[21]; Zambrana et al., 2012[24]). The HC participants included 288 males and 329 females; the gender of 247 participants was not stated. The mean age of the HC cohort was 58.25 (47-65) years. Among the HC participants, 165 were put forth as smokers while 434 were classified as non-smokers with the reminder (n = 265) unreported. The overall characteristics and data of the included studies are illustrated in Table 1(Tab. 1). 

Ten of the above studies stated their recruitment criteria for the control group. All studies provided assurances that these participants had received colonoscopies; however, some studies stated that these had been performed within the last two years while others did not report a time range (Nakhleh et al., 2017[13]). Six studies excluded patients with inflammatory bowel diseases, malignancies and those who had undergone recent gastrointestinal surgery or had a history of chemo or radiotherapy (Altomare et al., 2013[1], 2016[2]; Amal et al., 2016[3]; Nakhleh et al., 2017[13]; Sonoda et al., 2011[19]; Van Keulen et al., 2020[21]). Conversely, one study was more specific and excluded those who had an active infection and liver disease (Markar et al., 2019[12]), while another study excluded those who had mental health or who were pregnant (Wang et al., 2014[22]). 

Concerning breath sampling and collection methods, most studies collected these samples indirectly through collection bags that were subsequently transferred into vacuums, syringes, or glass vials except for three studies that had patients exhale directly into analyser systems (De Vietro et al., 2020[6]; Van Keulen et al., 2020[21]; Zambrana et al., 2012[24]). However, breath samples were collected under different specified conditions. Eight of these studies specified that participants were required to fast prior to sampling, ranging from 2-12 h (Altomare et al., 2013[1], 2016[2]; Amal et al., 2016[3]: De Vietro et al., 2020[6]; Markar et al., 2019[12]; Nakhleh et al., 2017[13]; Wang et al., 2014[22]; Zambrana et al., 2012[24]). Two studies prohibited smoking 2 h before sampling (Amal et al., 2016[3]; Nakhleh et al., 2017[13]). Another study required 12 h of withholding the ingestion of alcohol and coffee (Peng et al., 2010[15]). Three studies did not report such conditions. Moreover, one study had a protocol stated for excluding inadequate breath samples while others did not (Van Keulen et al., 2020[21]). Another condition to consider was the environment in which the breath sample was taken. Five of these studies disclosed that breath samples had been taken in the same area (Altomare et al., 2013[1]; De Vietro et al., 2020[6]; Markar et al., 2019[12]; Peng et al., 2010[15]; Van Keulen et al., 2020[21]), two of which kept participants in the room for at least 10 to 20 min before breath collection (Altomare et al., 2013[1]; Markar et al., 2019[12]) and one study had the participants' breath filtered by medical air (De Vietro et al., 2020[6]). Other factors included breath sample filtration and the quality of the exhaled breath. Four studies filtered residual environmental contaminants from the breath samples via flushing with nitrogen gas (Wang et al., 2014[22]) and by installing a filter (charcoal, carbon and bacterial) cartridge (Nakhleh et al., 2017[13]; Peng et al., 2010[15]; Van Keulen et al., 2020[21]). Concerning the quality of the breath samples, two studies had participants repeatedly inhaling for 3-5 min to reach total lung capacity (Amal et al., 2016[3]; Peng et al., 2010[15]). Other studies instructed participants to simply take a single deep nasal inhalation (n = 1) and to take five consecutive breaths (n = 1) (Markar et al., 2019[12]; Van Keulen et al., 2020[21]). One study collected breath samples specifically when the carbon dioxide level exceeded 3 % (Altomare et al., 2016[2]). Finally, only one study declared that breath samples were taken from CRC patients prior to surgery (Markar et al., 2019[12]). 

In relation to the analytical platforms, eight studies used Gas chromatography-mass spectrometry (GC-MS); three of these studies coupled GC-MS with thermal desorption (TD-GC-MS) (Altomare et al., 2013[1]; Depalma et al., 2014[7]; Di Lena et al., 2012[8]), while two studies integrated solid-phase micro-extraction (SPME-GC-MS) (Peng et al., 2010[15]; Wang et al., 2014[22]). The remaining studies utilized other analytical platforms including electronic nose (n = 2) (Altomare et al., 2016[2]; Van Keulen et al., 2020[21]), canine scent (n = 1) (Sonoda et al., 2011[19]), secondary electrospray ionisation (SESI-MS) (n = 1) (Zambrana et al., 2012[24]), selected ion flow tube (SIFT-MS) (n = 1) (Markar et al., 2019[12]) and a cross-reactive nanomaterial-based sensor with gold nanoparticles (GNP) (n = 1) (Leja et al., 2015[11]). Additionally, one study compared the performance of SPME-GC-MS against functionalized GNPs (Peng et al., 2010[15]). 

Other sources of variability included the different methods and protocols that the studies employed to classify and analyze VOCs in the breath samples. The leave-out method was employed by eight studies and these were further classified by either the support vector machine method or linear discriminant analysis (Altomare et al., 2013[1], 2016[2]; Amal et al., 2016[3]; Depalma et al., 2014[7]; Di Lena et al., 2012[8]; Nakhleh et al., 2017[13]; Van Keulen et al., 2020[21]; Zambrana et al., 2012[24]). The remaining studies used different methods as follows: pattern recognition methods (n = 3) (Amal et al., 2016[3]; Leja et al., 2015[11]; Nakhleh et al., 2017[13]), principal component analysis (n = 3) (Altomare et al., 2016[2]; Peng et al., 2010[15]; Wang et al., 2014[22]), a probabilistic neural network (n = 2) (Altomare et al., 2013[1], 2016[2]) and external VOC databases (n = 3) (De Vietro et al., 2020[6]; Wang et al., 2014[22]; Zambrana et al., 2012[24]). Moreover, some studies utilized artificial intelligence to run a predictive algorithm module that selected a small set of VOCs. Another factor that contributed to inconsistency was the length of time it took to analyze the breath samples from the time of breath collection. One study stated that samples were analyzed within 1 h (n = 1) (Markar et al., 2019[12]) after collection, whereas another study analyzed the samples four days after collection (Peng et al., 2010[15]). The remainder of the studies did not disclose these details. 

Quality assessment tests were performed on 10 of the included studies, four studies were excluded due to publication type (i.e. abstracts/posters) (Depalma et al., 2014[7]; Di Lena et al., 2012[8]; Leja et al., 2015[11]; Zambrana et al., 2012[24]). The STARD scores for the included articles ranged from 10 to 24 with an average of 17.7. The final scores of the STARD for each study are shown in Table 1(Tab. 1) and the full details of the scoring process are provided in the supplementary information, Appendix D. The QUADAS-2 test revealed that patient selection may have been a major source of bias because all studies were indicated as having had a high or unclear risk; this was due to the failure of providing recruitment criteria and due to the absence of a positive control group. Likewise, the absence of a validation set for the index test in selected studies, as well as the overall lack of clarity regarding the flow and timing also suggested a risk of bias. Consequently, the overall applicability quality of the included studies was high; however, the studies were at high risk of including bias. The QUADAS-2 tool results are summarized in Table 2(Tab. 2) (References in Table 2: Altomare et al., 2013[1], 2016[2]; Amal et al., 2016[3]; De Vietro et al., 2020[6]; Depalma et al., 2014[7]; Di Lena et al., 2012[8]; Leja et al., 2015[11]; Markar et al., 2019[12]; Nakhleh et al., 2017[13]; Peng et al., 2010[15]; Sonoda et al., 2011[19]; Van Keulen et al., 2020[21]; Wang et al., 2014[22]; Zambrana et al., 2012[24]).

### Meta-analysis

**Figure 3:**
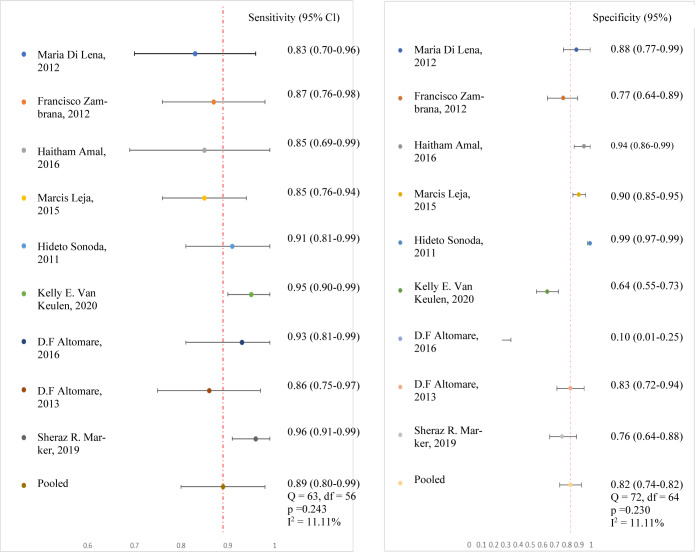
A forest plot of the sensitivities and specificities calculated using the second approach CI= Confidence Interval, Q= Cochran Chi-squared statistic, df= Degrees of freedom , and I^2^= Inconsistency calculated by 100 % × (Q - df)/Q

Subgroup meta-analysis was performed using both statistical methods that were previously described and the results are shown in Table 3(Tab. 3) (References in Table 3: Altomare et al., 2013[1], 2016[2]; Amal et al., 2016[3]; Di Lena et al., 2012[8]; Leja et al., 2015[11]; Markar et al., 2019[12]; Sonoda et al., 2011[19]; Van Keulen et al., 2020[21]; Zambrana et al., 2012[24]). Using the first approach, the pooled mean sensitivity and specificity of nine studies was calculated as 0.89 and 0.76, respectively (Altomare et al., 2013[1], 2016[2]; Amal et al., 2016[3]; Di Lena et al., 2012[8]; Leja et al., 2015[11]; Markar et al., 2019[12]: Sonoda et al., 2011[19]; Van Keulen et al., 2020[21]; Zambrana et al., 2012[24]). Using the second approach, the estimated diagnostic accuracy was measured using sensitivity, specificity, PPV, NPV, positive likelihood ratio (PLR), negative likelihood ratio (NLR) and the diagnostic odds ratio (DOR). Pooled sensitivity was 0.89 (95 % CI = 0.80-0.99) and specificity was 0.83 (95 % CI = 0.74-0.92). Heterogeneity in the pooled sensitivity was low at I^2 ^= 11.11 %, P = 0.243, while the heterogeneity of the pooled results for specificity was also low at I^2 ^= 11.11 %, P = 0.230. Mean PPV, mean NPV, PLR, NLR and DOR were 0.76 (95 % CI = 0.63-0.88), 0.93 (95 % CI = 0.86-0.99), 5.22 (95 % CI = 3.03-8.99), 0.13 (95 % CI = 0.05-0.32) and 42.95 (95 % CI = 12.67-145.62), respectively. A forest plot of the sensitivities and specificities calculated using the second approach was created to illustrate the results obtained in this study (see Figure 3(Fig. 3)). 

### The performance of individual volatile organic compounds

The individual VOCs detected in the breath of CRC patients were reported in nine studies with a combined total of 66. A quantitative summary of the VOCs that were found in at least three articles or more is illustrated in Table 4(Tab. 4) (References in Table 4: Altomare et al., 2013[1]; Amal et al., 2016[3]; De Vietro et al., 2020[6]; Leja et al., 2015[11]; Markar et al., 2019[12]; Nakhleh et al., 2017[13]; Peng et al., 2010[15]; Wang et al., 2014[22]; Zambrana et al., 2012[24]). The total number of CRC patients tested from the relevant articles was calculated to weigh the compound's significance. Ethanol had the highest significance as it was reported by four studies with a total of 204 CRC breath samples (Amal et al., 2016[3]; Leja et al., 2015[11]; Markar et al., 2019[12]; Nakhleh et al., 2017[13]). Acetone was detected in the breath of 161 samples from four studies in total (Amal et al., 2016[3]; De Vietro et al., 2020[6]; Leja et al., 2015[11]; Nakhleh et al., 2017[13]). Three studies with a combined 154 participants found ethyl acetate (Amal et al., 2016[3]; Leja et al., 2015[11]; Nakhleh et al., 2017[13]). Likewise, 4-methyloctane and nonanal were reported in three studies with 120 (Altomare et al., 2013[1]; Amal et al., 2016[3]; Leja et al., 2015[11]) and 115 (Altomare et al., 2013[1]; De Vietro et al., 2020[6]; Nakhleh et al., 2017[13]) participants, respectively. An additional comparison was made to discover how many of the most frequently reported compounds were noted in each study. The studies conducted by Amal (2016[3]) and Leja (2015[11]) reported ethanol, acetone, ethyl acetate and 4-methyloctane (Amal et al., 2016[3]; Leja et al., 2015[11]). The study conducted by Nakhleh (et al., 2017[13]) reported ethanol, acetone, ethyl acetate and nonanal (Nakhleh et al., 2017[13]). These three studies employed GC-MS. A complete and detailed table of all the reported VOCs is provided in the supplementary information, Appendix E.

## Discussion

An emerging number of studies have demonstrated the potential value of exhaled VOCs as a diagnostic and triaging test for CRC; however, their use in clinical practice is yet to be observed (Altomare et al., 2013[1], 2016[2]; Amal et al., 2016[3]; De Vietro et al., 2020[6]; De Palma et al., 2014[7]; Di Lena et al., 2012[8]; Leja et al., 2015[11]; Markar et al., 2019[12]; Nakhleh et al., 2017[13]; Peng et al., 2010[15]; Sonoda et al., 2011[19]; Van Keulen et al., 2020[21]; Wang et al., 2014[22]; Zambrana et al., 2012[24]). A study conducted by Altomare showed that the pattern of VOCs changed following the removal of CRC, thereby confirming a close relationship between tumor metabolism and exhaled VOCs (Altomare et al., 2013[1]). Similarly, a study conducted by Wang was able to detect nine VOCs with increased concentration in breath samples of CRC patients (Wang et al., 2014[22]). Additional research was able to identify higher levels of acetone and ethyl acetate in CRC patients (Amal et al., 2016[3]). More recently, in a study conducted by Sonoda, dogs were trained to distinguish CRC from healthy controls using exhaled breath samples with 91 % sensitivity and 95 % specificity results (Sonoda et al., 2011[19]). Multiple analytical platforms are used when attempting to analyze VOCs. GC-MS allows for volatile molecules to be physically separated and identified and for the exact composition of the sample to be determined. SPME can be utilised for sample pre-concentration when using GC-MS (Reade, 2016[17]). In this process, polymer-coated fiber is used to absorb compounds from patient samples. Other methods of extraction that utilize mass spectrometry include SESI-MS and SIFT-MS, and cross-reactive nanomaterial-based sensor with GNPs (Reade, 2016[17]).

This paper provides the first comprehensive and up-to-date review of the performance of exhaled VOCs in CRC detection. The given amalgamated results of the meta-analysis support the general hypothesis with a pooled sensitivity of 0.89 (95 % CI = 0.80-0.99) and specificity of 0.83 (95 % CI = 0.74-0.92). In addition, mean PPV and mean NPV were 0.76 (95 % CI = 0.63-0.88) and 0.93 (95 % CI = 0.86-0.99), respectively. These results imply that VOCs can be used to differentiate between patients with CRC relatively accurately, or at the very least, reliably differentiate healthy patients from those with a gastrointestinal pathology. The DOR is a single indicator of test accuracy; in this review, its result was 42.95 (95 % CI = 12.67-145.62) implying a good discriminatory test performance. The PLR was 5.5 (95 % CI = 3.03-8.99) indicating that a CRC patient is 5.5 times more likely to have a positive result when compared with a healthy patient. Similarly, the NLR was 0.13 (95 % CI = 0.05-0.32) implying that a patient with a negative result had a 9.7-fold decrease in the odds of having CRC. The heterogeneity of the included articles was I^2 ^= 11.11 % for pooled sensitivity and specificity, which suggested a low level of heterogeneity. Selected interpretations can be made from these results. That is, VOC analysis can be used to screen for CRC but it is not yet sufficiently conclusive to diagnose the condition on its own. Its use prior to invasive colonoscopies may, however, reduce the number of false positives in screening programs. 

### Limitations 

There were limited published studies on the subject and those that were included were either case-control or cross-sectional studies that included small participant samples ranging from 27 to 194 which reduces the statistical impact of the results. The selection criteria for the healthy controls' cohort differed between studies; some included only those with a completely negative colonoscopy while others included those with adenomas, bowel diseases or did not disclose such information. Similarly, these studies included CRC patients in different stages of the disease. Furthermore, the impact of factors such as age, gender and smoking status remained undetermined because a separate analysis in this regard was not reported by the studies. Studies varied significantly in terms of the collecting, handling, storing and analysis of their samples. Additionally, the instructions and protocols provided before collecting a sample varied between studies. Although the same breath collection bags were used in ten of these studies their storing time varied. Three studies that used the GC-MS method detected the four most common VOCs (Amal et al., 2016[3]; Leja et al., 2015[11]; Nakhleh et al., 2017[13]). The types of VOCs detected could be dependent on the analytical methods and processing techniques used, which dictates that the findings are partial to that. 

### Future development 

**Figure 4:**
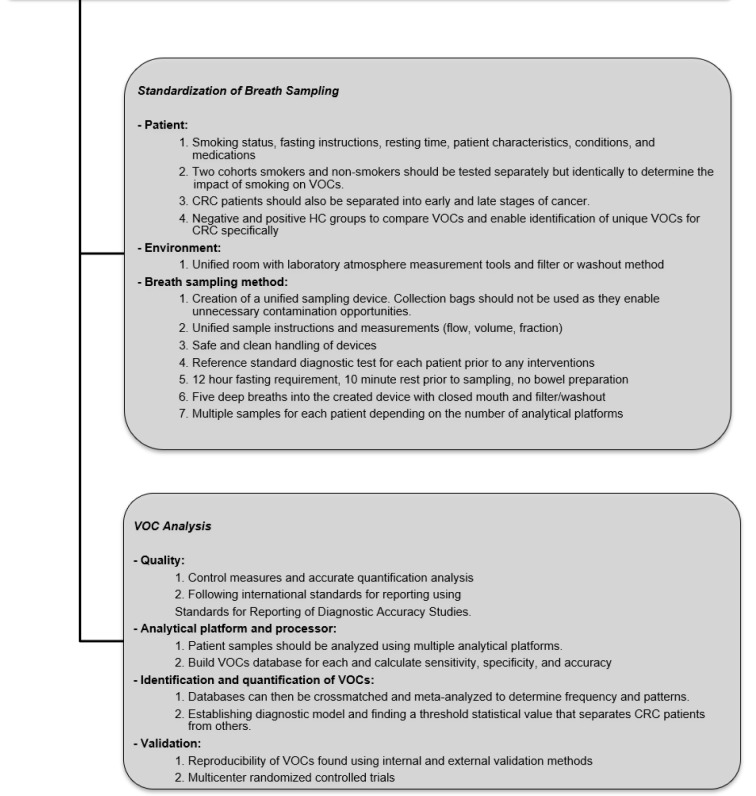
Proposed framework for conducting and reporting future studies evaluating the role of VOCs in CRC detection

The standardization of future research is warranted for ensuring its progression and advancement into clinical practice. To achieve this, extensive and well-controlled comparison studies are required to determine the superior analytical platform, processor and collection method. Confounding heterogeneity factors also require further study, particularly smoker vs non-smoker breath samples. Future studies must use larger sample sizes to confirm reliability. A proposed framework for conducting and reporting future studies on VOC breath testing in CRC patients was constructed, based on existing studies and the findings of this review in conjunction with STARD and QUADAS-2 checklists. The framework is illustrated in Figure 4(Fig. 4).

## Conclusion

Breath analysis is a time-efficient, inexpensive, safe, painless, and non-invasive method that is likely to have high patient compliance rates. However, the small sample size, the inconsistency of data and different analytical platforms currently limit the advancement of this field. It is predicted that, with research progression and standardization, breath analysis can eventually be used as an alternative and non-invasive mass screening tool prior to conducting colonoscopies, thereby reducing the number of patients undergoing colonoscopies and lowering FP rates accordingly. 

## Notes

We declare that our abstract was presented at a conference as an e-poster and was subsequently published on BJS as seen below: 

EP-575. Alsaadi D, Clements N, Gabuniya N, Chand M, Francis N. Exhaled volatile organic compounds in the detection of colorectal cancer: A systematic seview and meta-analysis. Brit J Surg 2002;109 (Suppl 5):znac245.139. doi: 10.1093/bjs/znac245.139. Published: 09 August 2022.

## Declaration

### Author contributions

Daniah Alsaadi - conceptualization, data collection and analysis, writing and reviewing.

Nicolle Clements; Natiya Gabuniya - data collection and analysis, reviewing.

Nader Francis; Manish Chand - Supervision and reviewing.

### Acknowledgments 

Not applicable.

### Conflict of interest 

The authors have no conflict of interest to disclose. 

### Funding 

The authors have no relevant financial or non-financial interests to disclose.

### Ethical approval and consent to participate

Ethical approval from the National Office for Research Ethics Committees and the University College London Guidance was not required.

## Supplementary Material

Supplementary information

## Figures and Tables

**Table 1:**
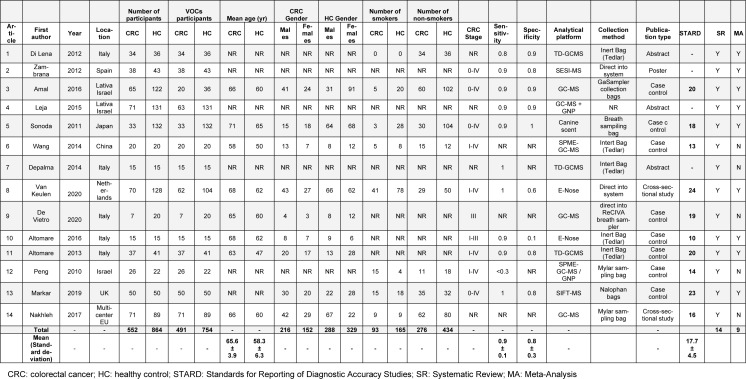
Overall characteristics, data, and STARD scores

**Table 2:**
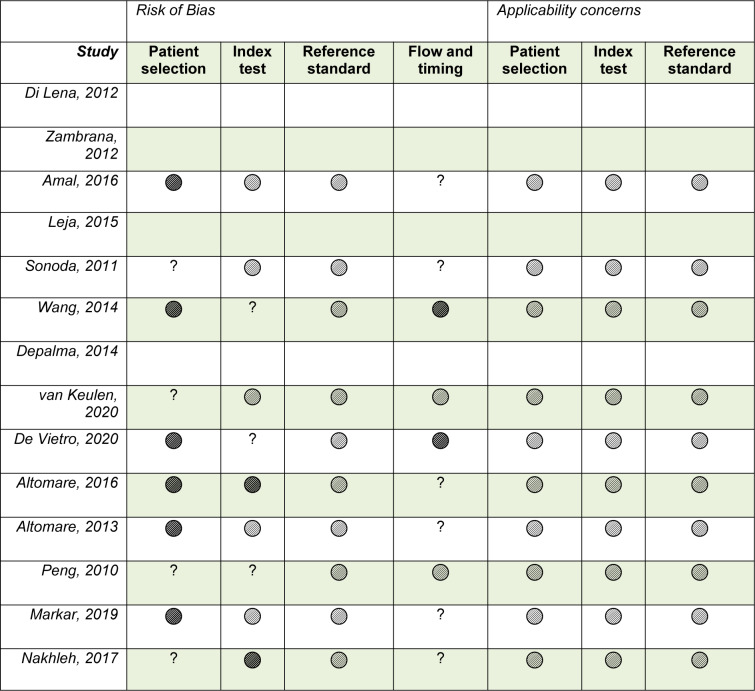
The QUADAS-2 tool

**Table 3:**
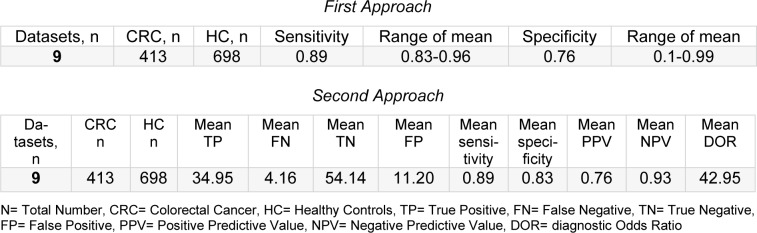
Subgroup analysis on exhaled VOCs based on two statistical approaches

**Table 4:**
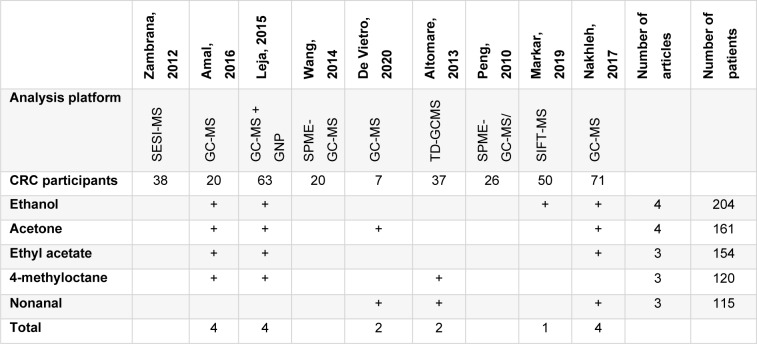
A quantitative summary of the commonly reported VOCs compounds
