# Supplementary material for: Exhaled volatile organic compounds in the detection of colorectal cancer: a systematic review and meta-analysis
Source: EXCLI J. 2024 May 17;23:795–810. doi: 10.17179/excli2024-7042 (PMC11231457; doi:10.17179/excli2024-7042)
Supplement: Supplementary information [file EXCLI-23-795-s-001.pdf]

EXCLI Journal 2024;23:795-810 – ISSN 1611-2156
Received: January 30, 2024, accepted: April 23, 2024, published: May 17, 2024

Supplementary information to:

Review article:

EXHALED VOLATILE ORGANIC COMPOUNDS IN THE DETECTION OF COLORECTAL CANCER: A SYSTEMATIC REVIEW AND META-ANALYSIS

Daniah Alsaadi[a,b], Nicolle Clements[b], Natiya Gabuniya[a,c], Nader Francis[a,d], Manish Chand[a]

a The Division of Surgery and Interventional Science, University College London, London, United Kingdom
b Clinical Research Facility Galway, Galway University Hospital, National University of Ireland, Galway, Republic of Ireland
c Plastic and Reconstructive Surgery Department, Guy's and St Thomas' Hospital, London, United Kingdom
d Department of General Surgery, Yeovil District Hospital NHS Foundation Trust, Yeovil, United Kingdom

* **Corresponding author:** Daniah Alsaadi, The Division of Surgery and Interventional Science, University College London, London, United Kingdom.
E-mail: daniah.alsaadi@outlook.com

https://dx.doi.org/10.17179/excli2024-7042

This is an Open Access article distributed under the terms of the Creative Commons Attribution License (http://creativecommons.org/licenses/by/4.0/).

APPENDIX A

Section and Topic	Item #	Checklist item	Location where item is reported
**TITLE**			
Title	1	Identify the report as a systematic review.	1
**ABSTRACT**			
Abstract	2	See the PRISMA 2020 for Abstracts checklist.	1
**INTRODUCTION**			
Rationale	3	Describe the rationale for the review in the context of existing knowledge.	2
Objectives	4	Provide an explicit statement of the objective(s) or question(s) the review addresses.	2
**METHODS**			
Eligibility criteria	5	Specify the inclusion and exclusion criteria for the review and how studies were grouped for the syntheses.	2-3
Information sources	6	Specify all databases, registers, websites, organisations, reference lists and other sources searched or consulted to identify studies. Specify the date when each source was last searched or consulted.	2-3
Search strategy	7	Present the full search strategies for all databases, registers and websites, including any filters and limits used.	3
Selection process	8	Specify the methods used to decide whether a study met the inclusion criteria of the review, including how many reviewers screened each record and each report retrieved, whether they worked independently, and if applicable, details of automation tools used in the process.	3
Data collection process	9	Specify the methods used to collect data from reports, including how many reviewers collected data from each report, whether they worked independently, any processes for obtaining or confirming data from study investigators, and if applicable, details of automation tools used in the process.	3-4
Data items	10a	List and define all outcomes for which data were sought. Specify whether all results that were compatible with each outcome domain in each study were sought (e.g. for all measures, time points, analyses), and if not, the methods used to decide which results to collect.	3-4
	10b	List and define all other variables for which data were sought (e.g. participant and intervention characteristics, funding sources). Describe any assumptions made about any missing or unclear information.	3-4
Study risk of bias assessment	11	Specify the methods used to assess risk of bias in the included studies, including details of the tool(s) used, how many reviewers assessed each study and whether they worked independently, and if applicable, details of automation tools used in the process.	3-4
Effect measures	12	Specify for each outcome the effect measure(s) (e.g. risk ratio, mean difference) used in the synthesis or presentation of results.	3-4
Synthesis methods	13a	Describe the processes used to decide which studies were eligible for each synthesis (e.g. tabulating the study intervention characteristics and comparing against the planned groups for each synthesis (item #5)).	3-4
	13b	Describe any methods required to prepare the data for presentation or synthesis, such as handling of missing summary statistics, or data conversions.	3-4
	13c	Describe any methods used to tabulate or visually display results of individual studies and syntheses.	3-4
	13d	Describe any methods used to synthesize results and provide a rationale for the choice(s). If meta-analysis was performed, describe the model(s), method(s) to	3-4

Section and Topic	Item #	Checklist item	Location where item is reported
		identify the presence and extent of statistical heterogeneity, and software package(s) used.	
	13e	Describe any methods used to explore possible causes of heterogeneity among study results (e.g. subgroup analysis, meta-regression).	3-4
	13f	Describe any sensitivity analyses conducted to assess robustness of the synthesized results.	3-4
Reporting bias assessment	14	Describe any methods used to assess risk of bias due to missing results in a synthesis (arising from reporting biases).	3-4
Certainty assessment	15	Describe any methods used to assess certainty (or confidence) in the body of evidence for an outcome.	3-4
**RESULTS**			
Study selection	16a	Describe the results of the search and selection process, from the number of records identified in the search to the number of studies included in the review, ideally using a flow diagram.	4-11
	16b	Cite studies that might appear to meet the inclusion criteria, but which were excluded, and explain why they were excluded.	4-11
Study characteristics	17	Cite each included study and present its characteristics.	4-11
Risk of bias in studies	18	Present assessments of risk of bias for each included study.	4-11
Results of individual studies	19	For all outcomes, present, for each study: (a) summary statistics for each group (where appropriate) and (b) an effect estimate and its precision (e.g. confidence/credible interval), ideally using structured tables or plots.	4-11
Results of syntheses	20a	For each synthesis, briefly summarise the characteristics and risk of bias among contributing studies.	4-11
	20b	Present results of all statistical syntheses conducted. If meta-analysis was done, present for each the summary estimate and its precision (e.g. confidence/credible interval) and measures of statistical heterogeneity. If comparing groups, describe the direction of the effect.	4-11
	20c	Present results of all investigations of possible causes of heterogeneity among study results.	4-11
	20d	Present results of all sensitivity analyses conducted to assess the robustness of the synthesized results.	4-11
Reporting biases	21	Present assessments of risk of bias due to missing results (arising from reporting biases) for each synthesis assessed.	13
Certainty of evidence	22	Present assessments of certainty (or confidence) in the body of evidence for each outcome assessed.	4-11
**DISCUSSION**			
Discussion	23a	Provide a general interpretation of the results in the context of other evidence.	11-13
	23b	Discuss any limitations of the evidence included in the review.	11-13
	23c	Discuss any limitations of the review processes used.	11-13
	23d	Discuss implications of the results for practice, policy, and future research.	11-13

EXCLI Journal 2024;23:795-810 – ISSN 1611-2156
Received: January 30, 2024, accepted: April 23, 2024, published: May 17, 2024

Section and Topic	Item #	Checklist item	Location where item is reported
**OTHER INFORMATION**			
Registration and protocol	24a	Provide registration information for the review, including register name and registration number, or state that the review was not registered.	2-3
	24b	Indicate where the review protocol can be accessed, or state that a protocol was not prepared.	2-3
	24c	Describe and explain any amendments to information provided at registration or in the protocol.	2-3
Support	25	Describe sources of financial or non-financial support for the review, and the role of the funders or sponsors in the review.	16
Competing interests	26	Declare any competing interests of review authors.	16
Availability of data, code and other materials	27	Report which of the following are publicly available and where they can be found: template data collection forms; data extracted from included studies; data used for all analyses; analytic code; any other materials used in the review.	2-3

EXCLI Journal 2024;23:795-810 – ISSN 1611-2156
Received: January 30, 2024, accepted: April 23, 2024, published: May 17, 2024

MOOSE (Meta-analyses Of Observational Studies in Epidemiology) Checklist

A reporting checklist for Authors, Editors, and Reviewers of Meta-analyses of Observational Studies. You must report the page number in your manuscript where you consider each of the items listed in this checklist. If you have not included this information, either revise your manuscript accordingly before submitting or note N/A.

Reporting Criteria	Reported (Yes/No)	Reported on Page No.
**Reporting of Background**		
Problem definition	Yes	1-2
Hypothesis statement	Yes	1-2
Description of Study Outcome(s)	Yes	1-2
Type of exposure or intervention used	Yes	1-2
Type of study design used	Yes	1-2
Study population	Yes	1-2
**Reporting of Search Strategy**		
Qualifications of searchers (eg, librarians and investigators)	Yes	3-4
Search strategy, including time period included in the synthesis and keywords	Yes	3-4
Effort to include all available studies, including contact with authors	Yes	3-4
Databases and registries searched	Yes	3-4
Search software used, name and version, including special features used (eg, explosion)	Yes	3-4
Use of hand searching (eg, reference lists of obtained articles)	Yes	appendix b
List of citations located and those excluded, including justification	Yes	3-4
Method for addressing articles published in languages other than English	Yes	3-4
Method of handling abstracts and unpublished studies	Yes	3-4
Description of any contact with authors	No	
**Reporting of Methods**		
Description of relevance or appropriateness of studies assembled for assessing the hypothesis to be tested	Yes	2-6
Rationale for the selection and coding of data (eg, sound clinical principles or convenience)	Yes	2-6
Documentation of how data were classified and coded (eg, multiple raters, blinding, and interrater reliability)	Yes	2-6
Assessment of confounding (eg, comparability of cases and controls in studies where appropriate	Yes	2-6

EXCLI Journal 2024;23:795-810 – ISSN 1611-2156
Received: January 30, 2024, accepted: April 23, 2024, published: May 17, 2024

Reporting Criteria	Reported (Yes/No)	Reported on Page No.
Assessment of study quality, including blinding of quality assessors; stratification or regression on possible predictors of study results	Yes	2-6
Assessment of heterogeneity	Yes	2-6
Description of statistical methods (eg, complete description of fixed or random effects models, justification of whether the chosen models account for predictors of study results, dose-response models, or cumulative meta-analysis) in sufficient detail to be replicated	Yes	2-6
Provision of appropriate tables and graphics	Yes	2-6
**Reporting of Results**		
Table giving descriptive information for each study included	Yes	6-11
Results of sensitivity testing (eg, subgroup analysis)	Yes	6-11
Indication of statistical uncertainty of findings	Yes	6-11
**Reporting of Discussion**		
Quantitative assessment of bias (eg, publication bias)	Yes	12-13
Justification for exclusion (eg, exclusion of non–English-language citations)	Yes	6-11
Assessment of quality of included studies	Yes	6-11
**Reporting of Conclusions**		
Consideration of alternative explanations for observed results	Yes	14
Generalization of the conclusions (ie, appropriate for the data presented and within the domain of the literature review)	Yes	14
Guidelines for future research	Yes	14
Disclosure of funding source	Yes	14

EXCLI Journal 2024;23:795-810 – ISSN 1611-2156
Received: January 30, 2024, accepted: April 23, 2024, published: May 17, 2024

APPENDIX B

Full list of search string used:

Connection	Word
	Exhal* [Text Word]
OR	Exhal* [MESH terms]
OR	Exhal* [Title/Abstract]
OR	Breathomics [Text Word]
OR	Breathomics [MESH terms]
OR	Breathomics [Title/Abstract]
OR	Respiratory [Text Word]
OR	Respiratory [MESH terms]
OR	Respiratory [Title/Abstract]
OR	Expiratory [Text Word]
OR	Expiratory [MESH terms]
OR	Expiratory [Title/Abstract]
OR	Odour [Text Word]
OR	Odour [MESH terms]
OR	Odour [Title/Abstract]
OR	Odor [Text Word]
OR	Odor [MESH terms]
OR	Odor [Title/Abstract]
OR	Sniff* [Text Word]
OR	Sniff* [MESH terms]
OR	Sniff* [Title/Abstract]
OR	Nose [Text Word]
OR	Nose [MESH terms]
OR	Nose [Title/Abstract]
OR	E-nose [Text Word]
OR	E-nose [MESH terms]
OR	E-nose [Title/Abstract]
OR	Electronic-nose [Text Word]
OR	Electronic-nose [MESH terms]
OR	Electronic-nose [Title/Abstract]
OR	Olfaction [Text Word]
OR	Olfaction [MESH terms]
OR	Olfaction [Title/Abstract]
OR	Scent [Text Word]
OR	Scent [MESH terms]
OR	Scent [Title/Abstract]
OR	Canine [Text Word]
OR	Canine [MESH terms]
OR	Canine [Title/Abstract]
**AND**	
	Volatile organic compound* [Text Word]
OR	Volatile organic compound* [MESH terms]
OR	Volatile organic compound* [Title/Abstract]

OR	VOC* [Text Word]
OR	VOC* [MESH terms]
OR	VOC* [Title/Abstract]
OR	Breath collecting apparatus [Text Word]
OR	Breath collecting apparatus [MESH terms]
OR	Breath collecting apparatus [Title/Abstract]
OR	BCA [Text Word]
OR	BCA [MESH terms]
OR	BCA [Title/Abstract]
OR	Gas chromatography [Text Word]
OR	Gas chromatography [MESH terms]
OR	Gas chromatography [Title/Abstract]
OR	Mass spectrometry [Text Word]
OR	Mass spectrometry [MESH terms]
OR	Mass spectrometry [Title/Abstract]
OR	GC [Text Word]
OR	GC [MESH terms]
OR	GC [Title/Abstract]
OR	MS [Text Word]
OR	MS [MESH terms]
OR	MS[Title/Abstract]
OR	Nanosensor [Text Word]
OR	Nanosensor [MESH terms]
OR	Nanosensor [Title/Abstract]
OR	Metabolomic [Text Word]
OR	Metabolomic [MESH terms]
OR	Metabolomic [Title/Abstract]
OR	Metabolites [Text Word]
OR	Metabolites [MESH terms]
OR	Metabolites [Title/Abstract]
**AND**	
	Colorectal Cancer [Text Word]
OR	Colorectal Cancer [MESH terms]
OR	Colorectal Cancer [Title/Abstract]
OR	CRC [Text Word]
OR	CRC [MESH terms]
OR	CRC [Title/Abstract]
OR	Colorectal malignan* [Text Word]
OR	Colorectal malignan* [MESH terms]
OR	Colorectal malignan* [Title/Abstract]
OR	Colorectal neoplasm* [Text Word]
OR	Colorectal neoplasm* [MESH terms]
OR	Colorectal neoplasm* [Title/Abstract]
OR	Colon Cancer [Text Word]
OR	Colon Cancer [MESH terms]
OR	Colon Cancer [Title/Abstract]
OR	CC [Text Word]

EXCLI Journal 2024;23:795-810 – ISSN 1611-2156
Received: January 30, 2024, accepted: April 23, 2024, published: May 17, 2024

OR	CC [MESH terms]
OR	CC [Title/Abstract]
OR	Rectal cancer [Text Word]
OR	Rectal cancer [MESH terms]
OR	Rectal cancer [Title/Abstract]
**OR**	RC [Text Word]
**OR**	RC [MESH terms]
**OR**	RC [Title/Abstract]

Asterisk symbol (*) was applied for truncation to enable different forms of a word to searched for simultaneously.

EXCLI Journal 2024;23:795-810 – ISSN 1611-2156
Received: January 30, 2024, accepted: April 23, 2024, published: May 17, 2024

APPENDIX C

Data extraction

A data abstraction template was created in Excel by the first author. Two investigators (DA and NC) independently extracted the following information from each eligible article where possible: (1) the title; (2) the first author indicated; (3) the study type; (4) the publication year; (5) the location where the study was based; (6) the number of CRC participants; (7) the number of HC participants; (8) the number of CRC participants sampled for VOC testing; (9) the number of HC participants sampled for VOC testing; (10) the mean age of the CRC group; (11) the mean age of the HC group; (12) the gender distribution numbers in the CRC group; (13) the gender distribution numbers in the HC group; (14) the percentage of smokers/number in the CRC group; (15) the percentage of smokers/number in the HC group; (16) CRC stage; (17) the relevant breath collection method and its steps; (18) analytical platform and classifier; (19) the VOC biomarkers detected and their chemical classes; (20) cut-off value; (21) the sensitivity, (22) specificity, (23) and recognition rates; (24) accuracy; (25) P-value; (26) the area under a curve; (27) gradient; (28) true positive (TP), false negative (FN), false positive (FP) and true negative (TN) values, as well as positive predictive value (PPV) and negative predictive value (NPV); (29) the eligibility criteria of each study. Minimal inconsistencies were found when a third and fourth reviewer (SN and CP) compared the two data extraction forms; these were rectified through revision and discussion.

APPENDIX D

STARD scoring:

		Maria Di Lena, 2012	Francisco Zambrana, 2012	Haitham Amal, 2016	Marcis Leja, 2015	Hideto Sonoda , 2011	Changsong Wang, 2014	N.Depalma, 2014	Kelly E. Van Keulen , 2020	Nicoletta De Vietro, 2020	D. F. Altomare, 2016	Altomare, 2013	G Peng, 2010	Sheraz R. Markar, 2019	Morad K. Nakhleh, 2017
**TITLE OR ABSTRACT**															
	1			1		1	0		1	0	0	1	0	1	0
**ABSTRACT**															
	2			1		1	1		1	0	0	1	1	1	0
**INTRODUCTION**															
	3			1		1	1		1	1	1	1	1	1	1
	4			0		0	0		1	1	1	1	0	1	0
**METHODS**															
**Study design**	5			1		1	1		1	1	1	1	1	1	1
**Participants**	6			1		1	1		1	1	0	0	0	1	0
	7			1		1	1		1	1	0	1	1	1	0
	8			0		1	0		0	0	0	1	0	0	1
	9			0		1	0		0	1	0	1	0	0	0
**Test methods**	10a			1		1	1		1	1	1	1	1	1	1
	10b			1		0	0		0	0	0	0	0	1	0
	11			1		0	1		0	1	1	1	1	1	0
	12a			0		0	0		0	1	0	0	0	0	0
	12b			1		1	1		1	0	0	1	1	1	1
	13a			1		0	1		1	0	1	1	1	1	1
	13b			0		0	0		0	0	0	0	0	0	0
**Analysis**	14			0		1	0		1	1	0	1	0	1	1
	15			0		1	0		1	1	0	0	0	0	0
	16			0		0	0		0	0	0	0	0	0	0
	17			1		1	0		1	1	0	0	1	0	1
	18			0		0	0		1	0	0	0	0	0	0
**RESULTS**															
**Participants**	19			0		0	0		1	0	0	1	0	0	0
	20			1		1	1		1	1	1	1	1	1	1
	21a			1		1	0		1	1	1	1	1	1	1
	21b			1		1	1		1	0	1	1	1	1	1
	22			0		0	0		0	0	0	0	0	0	0
**Test results**	23			0		1	0		1	1	0	1	0	1	0
	24			1		0	0		1	1	0	1	0	1	1
	25			0		0	0		0	0	0	0	0	0	0
**DISCUSSION**															
	26			1		0	0		1	1	0	0	0	1	1
	27			1		0	1		1	1	1	1	1	1	1
**OTHER INFORMATION**															
	28			1		0	0		1	0	0	0	0	1	0
	29			0		0	0		0	0	0	0	0	1	1
	30			1		1	1		1	1	0	0	1	1	1
**Total**				**20**		**18**	**13**		**24**	**19**	**10**	**20**	**14**	**23**	**16**

EXCLI Journal 2024;23:795-810 – ISSN 1611-2156
Received: January 30, 2024, accepted: April 23, 2024, published: May 17, 2024

APPENDIX E

Full list of VOCs detected:

	Zambrana, 2012	Amal, 2016	Leja, 2015	Wang, 2014	De Vietro, 2020	Altomare, 2013	Peng, 2010	Markar, 2020	Nakhleh, 2017	**Total**
**Ethanol**		+	+					+	+	**4**
**Acetone**		+	+		+				+	**4**
**4-Methyloctane**		+	+			+				**3**
**Ethyl acetate**		+	+						+	**3**
**Nonanal**					+	+			+	**3**
**Undecane**						+			+	**2**
**Dodecane**				+	+					**2**
**Benzene ethyl**					+				+	**2**
**Decanal**					+	+				**2**
**Styrene**			+						+	**2**
**2-Ethylhexanol**									+	**1**
**Valerenic acid**	+									**1**
**2-Oxo-4-phosphono-butanoate**	+									**1**
**DICAMBA (2,4-D di-chlorophenoxyacetic acid)**	+									**1**
**Kojic acid**	+									**1**
**Muconic acid**	+									**1**
**Phosphohydroxypy-ruvic acid**	+									**1**
**Cyclohexanone**				+						**1**
**2,2-Dimethyldecane**				+						**1**
**4-Ethyl-1-octyn-3-ol**				+						**1**
**Ethylaniline**				+						**1**
**Cyclooctylmethanol**				+						**1**
**Trans-2-dodecen-1-ol**				+						**1**
**3-Hydroxy-2,4,4-tri-methylpentyl 2- me-thylpropanoate (less)**				+						**1**

**Acetonitrile**					+					**1**
**Benzaldehyde**					+					**1**
**Benzene methyl**					+					**1**
**Butanoic acid**					+					**1**
**Diallyl disulfide**					+					**1**
**Dodecanoic acid**					+					**1**
**Eptanal**					+					**1**
**Hexanal**					+					**1**
**Indole**					+					**1**
**Octanoic acid**					+					**1**
**Pentanal**					+					**1**
**Pentanoic acid**					+					**1**
**Phenol**					+					**1**
**Propanoic acid**					+					**1**
**Tetradecane**					+					**1**
**4-Methyl-2-penta-none**						+				**1**
**2-Methylpentane 87 3-Methylpentane**						+				**1**
**2-Methylbutane**						+				**1**
**2-Methylpentane**						+				**1**
**Methylcyclopentane**						+				**1**
**Cyclohexane**						+				**1**
**Methylcyclohexane, 1,3-dimethylbenzene**						+				**1**
**1,4-Dimethylbenzene**						+				**1**
**Trimethyldecane**						+				**1**
**1,2-Pentadienee**						+				**1**
**2-Amino-5-isopropyl-8-methyl-1-azulene-carbonitrile**							+			**1**
**4-(4-Propylcyclo-hexyl)-4^{0} -cyano[1,1^{0} -biphenyl]-4-yl ester benzoic acid**							+			**1**
**(1,1-Dime-thylethyl)thio] acetic acid**							+			**1**

**1-Iodo nonane**							+			**1**
**1,3-Dimethyl benzene**							+			**1**
**1,10-(1-Butenyli-dene)bis benzene**							+			**1**
**Propanal**								+		**1**
**Carbon disulphide**								+		**1**
**Ammonia**								+		**1**
**Butanol**								+		**1**
**Propanol**								+		**1**
**Acrolein**								+		**1**
**3-Methylhexane**									+	**1**
**5-Ethyl-3-methyloctane**									+	**1**
**Isononane**									+	**1**
**Isoprene**									+	**1**
**Toluene**									+	**1**
**Total**	**6**	**4**	**5**	**8**	**20**	**14**	**6**	**7**	**13**	

EXCLI Journal 2024;23:795-810 – ISSN 1611-2156
Received: January 30, 2024, accepted: April 23, 2024, published: May 17, 2024

APPENDIX F

List of abbreviations

**Abbreviation**	**Term**
CRC	Colorectal Cancer
AJCC	The American Joint Committee on Cancer
TNM	Tumour, Node, Metastasis
BRFSS	The Behavioral Risk Factor Surveillance System
US	United States
UK	United Kingdom
gFOBT	Guaiac-Based Fecal Occult Blood Testing
FIT	Fecal Immunochemical Testing
Hb	Hemoglobin
VOCs	Volatile Organic Compounds
HC	Healthy Control
GC-MS	Gas Chromatography and Mass Spectrometry
TD-GC-MS	Gas Chromatography and Mass Spectrometry with Thermal Desorption
SPME-GC-MS	Solid-Phase Microextraction Gas Chromatography and Mass Spectrometry
SESI-MS	Electrospray Ionization Mass Spectrometry
SIFT-MS	Selected-Ion Flow-Tube Mass Spectrometry
GNP	Gold Nano-Particles
PRISMA	Preferred Reporting Items for Systematic Review and Meta-Analyses
MOOSE	Meta-Analysis of Observational Studies in Epidemiology
UCL	University College London
JS	Jacqui Smith
DA	Daniah Alsaadi
NC	Nicolle Clements
CP	Chee Lin Piong
TP	True Positive
TN	True Negative
FP	False Positive
FN	False Negative
PPV	Positive Predictive Value
NPV	Negative Predictive Value
SN	Sofia Nazir
QUADAS-2	The Quality Assessment of Diagnostic Accuracy Studies 2
STARD	Standards For Reporting of Diagnostic Accuracy Studies
CI	The Confidence Interval
PLR	Positive Likelihood Ratio
NLR	Negative Likelihood Ratio
DOR	Diagnostic Odds Ratio
I^2	Inconsistency
Q	Cochran Chi-squared Statistic
df	Degrees of freedom
